# Impact of new water systems on healthcare-associated colonization or infection with Pseudomonas aeruginosa

**DOI:** 10.3205/dgkh000272

**Published:** 2016-05-30

**Authors:** Annick Lefebvre, Catherine Quantin, Philippe Vanhems, Jean-Christophe Lucet, Xavier Bertrand, Karine Astruc, Pascal Chavanet, Ludwig S. Aho-Glélé

**Affiliations:** 1 Service d’épidémiologie et hygiène hospitalières, CHU Dijon, France; 2 Laboratoire Microbiologie Environnementale et Risques Sanitaires, Dijon, France; 3 Equipe opérationnelle d’hygiène, CHU Reims, Hôpital Maison Blanche, Reims, France; 4 Service de Biostatistiques et Information Médicale, CHU Dijon, France; 5 Département d’épidémiologie – EA 4184, Université de Bourgogne, Dijon, France; 6 Inserm UMR 1181 «Biostatistique, Biomathématique, PharmacoEpidémiologie et Maladies Infectieuses», Université de Bourgogne Franche-Comté, Dijon, France; 7 Service d’Hygiène Hospitalière, Epidémiologie et Prévention, groupe hospitalier Edouard Herriot, Lyon, France; 8 Equipe d’épidémiologie et santé publique, Université Claude Bernard, Lyon, France; 9 UHLIN, groupe hospitalier Bichat – Claude Bernard, HUPNVS, AP-HP, Paris, France; 10 Université Paris Diderot, Paris, France; 11 Service d’hygiène, CHU Besançon, France; 12 Laboratoire Chrono-environnement, UMR CNRS 6249, Université de Franche-Comté, Besançon, France; 13 Département de maladies infectieuses, CHU Dijon, France

**Keywords:** Pseudomonas aeruginosa, water systems, healthcare-associated infections, infection control

## Abstract

**Aim: **We aimed to study the impact of new water systems, which were less contaminated with *P. aeruginosa*, on the incidence of healthcare-associated *P. aeruginosa* cases (colonizations or infections) in care units that moved to a different building between 2005 and 2014.

**Methods:** Generalized Estimated Equations were used to compare the incidence of *P. aeruginosa* healthcare-associated cases according to the building.

**Results:** Twenty-nine units moved during the study period and 2,759 cases occurred in these units. No difference was observed when the new building was compared with older buildings overall.

**Conclusion:** Our results did not support our hypothesis of a positive association between water system contamination and the incidence of healthcare-associated *P. aeruginosa* cases. These results must be confirmed by linking results of water samples and patients’ data.

## Zusammenfassung

**Zielsetzung:** Untersucht werden sollte der Einfluss eines neuen weniger mit *P. aeruginosa* kontaminierten Wassersystems auf die Inzidenz nosokomialer Kolonisation oder Infektion mit *P. aeruginosa* in Pflegeeinheiten, die zwischen 2005 und 2014 in ein anderes Gebäude verlagert wurden.

**Methode:** Mit dem Modell der generalisierten Schätzgleichungen (Generalized Estimated Equations) wurde die Inzidenz nosokomialer Fällen von *P. aeruginosa* zwischen den beiden Gebäuden verglichen.

**Ergebnisse: **29 Einheiten mit 2.759 in diesen Einheiten nachgewiesenen Fällen wurden während des Studienzeitraums verlagert. Zwischen dem neuem und dem alten Gebäude war keine Differenz nachweisbar.

**Schlussfolgerung: **Die Ergebnisse unterstützen nicht die Hypothese einer positiven Assoziation zwischen der Trinkwasserkontamination und der Inzidenz nosokomialer *P. aeruginosa*-Fälle. Allerdings bedarf diese Aussage der Überprüfung des Zusammenhangs zwischen kontaminierten Wasserproben und Patientendaten.

## Introduction

The possible presence of *Pseudomonas aeruginosa* in water systems is well established [1], [2]. However, the link between contamination of the water system and hospital-acquired infections (HAI) is less clear [3]. Indeed, reports in the literature concern specific care units such as intensive care units (ICU), or neonatal units..., and they report outbreak investigations [4], [5]. The literature review of Anaissie et al. [4] published in 2002 provided a summary of infections other than *Legionella *spp. acquired from the hospital water supply. They found no large-scale, long-term studies. All reviewed studies concerned outbreaks linked to contamination of hospital water systems. Many of these studied the relationship between micro-organisms found in water systems and those identified in patients using genotyping methods. The causative organisms were mostly *P. aeruginosa*. A recent literature review [6] confirmed that all of these studies were conducted during epidemics. Even though the relationship between *P. aeruginosa* in water systems and hospital-acquired *P. aeruginosa* infections has not been studied thoroughly, such a relationship is considered plausible. Guidelines vary across countries and experts [3], [7]. The Centers for Disease Control and Prevention recommend sampling water if a water source is suspected or confirmed in patient HAI or in cases of clusters of *Legionella* [8]. This is contrary to French guidelines, which recommend systematic monitoring for *Legionella* and *P. aeruginosa* [9]. 

Dijon University Hospital experienced two waves of moves in 2010–2011, then in 2013–2014, with the construction of a new building and the renovation of a building in which the water system was completely renovated and structurally modified. Samples of water taken from various buildings were regularly contaminated with* P. aeruginosa*. In contrast, in the new buildings, contamination was exceptional, except for a few units. 

The objective of this study was to evaluate the impact of the building on the incidence of HAI or colonizations with *P. aeruginosa* in the care units that moved to a different building at least once between January 2005 and April 2014. The underlying assumption was that the move from old buildings to new ones would reduce exposure to water-borne contamination with *P. aeruginosa* and therefore decrease HAI or colonizations due to* P. aeruginosa*. Indeed, older water networks are expected to be more corroded and to have a more mature biofilm [10].

## Patients and methods

### Setting

The University Hospital of Dijon is located in Burgundy, France and has 1,800 beds, with medical and surgical units and ICUs. There are two main sites; the General Hospital (GH) constructed in the 18^th^ century is near the centre of Dijon, and the more recent Bocage site, which is in the suburbs of Dijon. The Bocage site comprises “Old building 1” (O1) made up of three buildings with few care units constructed in 1960, Old building (O2), Bocage 62 (B62), and the New Building (New B). B62 was constructed after O1 and O2 and had a new water system fitted during renovation work. It was therefore less contaminated with *P. aeruginosa* than were the others. B62 was grouped with the new building (NewB) after the move. Altogether, five buildings or groups of buildings (GH, O1, O2, B62 and New B) were involved in the moving of units from one building to another. The moves occurred during 2010–2011 after the construction of the NewB, and during 2013–2014 after the renovation work of B62. All moves, and not only moves from old to new buildings, were considered.

### Water samples

In our facility, water samples had to be taken and tested in each ward once every quarter, with nearly 20% of water outlets sampled. Water outlets were sampled randomly, except for water outlets in the nursing room, which have to be sampled at least once a year. Samples from water outlets with filters fitted were taken after removing the filter. Only the first sample in the same localization (same room, same water outlet) during the quarter was kept. Water samples taken from operating rooms were excluded.

### Patients

All *P. aeruginosa*-positive samples (pulmonary, skin, urine, blood…) in Dijon Hospital between January 2005 and April 2014 were extracted from the bacteriology laboratory database. Duplicates were defined on the basis of the antibiotype and a 6-month period and were excluded. Two isolates were considered different if they were isolated at more than six months apart or if a major difference of antibiotic resistance (one susceptible isolate, one resistant isolate) was observed for one of the following antibiotics: ticarcillin, piperacillin, ceftazidime, imipenem, meropenem, aztreonam, gentamicin, tobramycin, amikacin, ciprofloxacin, and colistin, according to Antimicrobial Committee of the French Society for Microbiology 2013 [11]. Patients were also excluded if they had been hospitalized for less than 48 hours at the time the sample was taken, and they were considered located in the unit where the procedure leading to the first positive sample was prescribed. Only units that were moved from one building to another during the study period were selected.

The number of person-days per month was obtained from administrative databases. The population at risk was calculated for each day by dividing the number of person-days in the month by the number of days in the month. The sum of patient-days and cases by building was calculated for each care unit and each year. 

### Statistical analyses

The spatial unit of analysis was the care unit, which typically includes 4 to 43 beds. We used Generalized Estimated Equations (GEE) with binomial negative regression of the number of *P. aeruginosa* cases (colonizations or infections), with patients-days as exposure variables to test the impact of the building on the rate of HAI or colonizations in the care unit. This model allowed us to take correlations between observations within each care unit into account. The time unit was the year. Person-days and *P. aeruginosa* cases were attributed to the previous year if a move occurred during the first half of the year, or to the next year if the move occurred in the second half of the year. A time trend was examined. The effect of the move was also tested (presence or absence of a move in the year). Sensitivity analyses were performed by removing ICUs from other units because all of the water outlets used for bathing patients in these wards have filters fitted and patients were thus regarded as unexposed to water contamination. Furthermore these units are a major source of HAI and may carry a high weight in analyses. The water samples collected within several units after the move were repeatedly contaminated with *P. aeruginosa*. Therefore, these units were subsequently excluded from the sensitivity analyses. A first-order autoregressive correlation matrix is generally suitable in GEE for repeated measurements of data in time [12]. This structure was used. The “robust” option allowed standard errors to overcome a poor specification of the correlation structure [13]. A QIC (Quasi-likelihood Information Criterion) was used to choose the model [14]. A p<0.05 was considered significant. Stata 10.0 software was used for the analyses [15].

## Results

After the removal of duplicates, 2932 water samples were studied for the period from 1^st^ July 2004 to 15^th^ February 2013. Among these, 493 or 16.8% (95% CI 15.5–18.2%) were positive for *P. aeruginosa*. Preliminary analyses of the results of water samples showed a lower proportion of *P. aeruginosa*-positive samples in the new building than in the others. Indeed, 4% of water samples were positive in the NewB versus 19% in older buildings (p<0.001). Twenty-nine units were involved in moves. Four units moved twice from one building to another. Between January 2005 and April 2014, 2,759 healthcare-associated cases (colonizations or infections) of *P. aeruginosa* occurred and 1,958,985 patient-days were recorded for the units concerned. The incidence was thus 141 per 100,000 person-days. The incidence increased over the years (coefficient of 0.043, p<0.001 in negative binomial regression). 

Six pairs of buildings were affected by the moves. The couples GH-NewB, and B62-NewB involved 12 and 13 care units, respectively; the couple O2-NewB involved 5 units. The other three couples involved only two or three care units each. 

The data showed under-dispersed Poisson distribution. The negative binomial regression was thus used. Moving in the year was not associated with the incidence of *P. aeruginosa*. Thus, this variable was removed from multivariate analyses. The results of the GEE model showed a lower incidence for GH than for NewB: IRR 0.69, p=0.039 (Table 1 (Tab. 1)). An overall effect of the building was observed (p<0.001). With the exclusion of units with positive water samples and ICUs, the results were similar. When the NewB was compared with all the others, no difference was observed. 

## Discussion

All cases of *P. aeruginosa* occurring during more than 9 years were included. This long-term study concerned 29 units cumulating 2,759 *P. aeruginosa* cases during 1,958,985 patient-days. 

The results of this study are not clearly in favour of our assumption that moving to a new building would lead to less exposure of patients to water contamination, and would thus cause a decrease in cases. A momentary disruption of the ward organisation at the time of the move could have increased the risk of HAI. However, no association was found with this variable. The probable longer-term reorganisation of wards after moving, with a lower caregiver-to-patient ratio, could also balance out the benefit of new water systems. Samples from the old buildings showed contamination. As a corrective measure, several water outlets had filters fitted, thus eliminating exposure to contaminated water. Conversely, in the NewB, as few samples had been taken because the move was recent, filters had been fitted at very few outlets. In addition, in the NewB, there are more water outlets so they were probably less frequently used than was the case in old buildings. This can lead to the constitution of a biofilm [16]. However, samples were mostly negative. Finally, several apparatus may have been reconnected to the new water supply (water fountains, dialysis equipment, endoscope washers…). These could favour the contamination of the new water system.

This study had some limitations. Firstly, the cases were assigned to the care unit that prescribed the sample, which was generally but not necessarily the care unit where the patient was located. Moreover, though we required a minimum of 48 hours of hospitalisation before the first positive sample to take account of that sample, the time the patient spent in the unit before the positive sample was not taken into account. However, patients rarely moved from one unit to another. Secondly, several care units moved in 2014. These recent moves led to a small number of patient-days for the NewB and thus lower power. Thirdly, person-days and *P. aeruginosa* cases were attributed to the previous year if a move occurred during the first half of the year, or to the next year if the move occurred in the second half of the year. This could lead to a bias in the estimation of the effect of the year, but the bias in the estimation of the building, which was the variable of interest, is probably very low. Shorter periods could have been used to minimize this bias but the power would have decreased. Fourthly, this retrospective study did not allow differentiating colonizations and infections. Finally, the absence of statistical association does not exclude the possibility that the water supply could serve as a reservoir for colonization or infection, which could not be evidenced by long-term surveillance. Furthermore, confounding factors which could not be taken into account (patient risk factors, caregiver-to-patient ratio…) may have biased the estimation of the effect of the building.

In conclusion, the results of this long-term study, which included 29 care units that moved from one building to another, did not support our hypothesis of an association between contamination of the water system and the incidence of HAI or colonizations with *P. aeruginosa*, outside particular situations such as epidemics. However, other confounding factors could counterbalance the benefits of the new water system. These include the long-term reorganisation with a lower caregiver/patient ratio or an increase in the number of water outlets, which are thus used less often. These results must be confirmed by studies that explore the association between contamination of the water system and clinical infections with *P. aeruginosa*, by linking the results of water samples and patients’ data using molecular biotyping.

## Notes

### Acknowledgments

We thank Philip Bastable for his editorial assistance.

### Competing interests

The authors declare that they have no competing interests.

## Figures and Tables

**Table 1:**
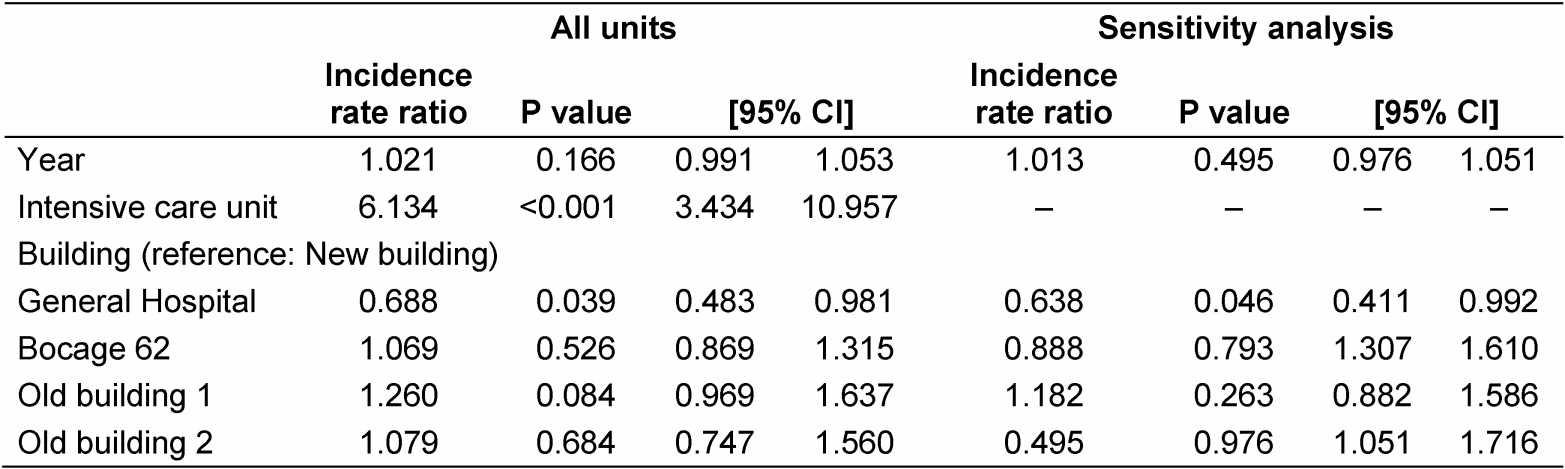
First-order auto-regressive GEE negative binomial models of number of cases of *P. aeruginosa*, multivariate analysis with hospitalisation days as offset, with and without intensive care units (sensitivity analysis)

## References

[1] Lavenir R, Sanroma M, Gibert S, Crouzet O, Laurent F, Kravtsoff J, Mazoyer MA, Cournoyer B (2008). Spatio-temporal analysis of infra-specific genetic variations among a Pseudomonas aeruginosa water network hospital population: invasion and selection of clonal complexes. J Appl Microbiol.

[2] Trautmann M, Lepper PM, Haller M (2005). Ecology of Pseudomonas aeruginosa in the intensive care unit and the evolving role of water outlets as a reservoir of the organism. Am J Infect Control.

[3] Decker BK, Palmore TN (2014). Waterborne pathogen detection: more than just "location, location, location...". Infect Control Hosp Epidemiol.

[4] Anaissie EJ, Penzak SR, Dignani MC (2002). The hospital water supply as a source of nosocomial infections: a plea for action. Arch Intern Med.

[5] Phillips MS, von Reyn CF (2001). Nosocomial infections due to nontuberculous mycobacteria. Clin Infect Dis.

[6] Loveday HP, Wilson JA, Kerr K, Pitchers R, Walker JT, Browne J (2014). Association between healthcare water systems and Pseudomonas aeruginosa infections: a rapid systematic review. J Hosp Infect.

[7] Exner M, Kramer A, Lajoie L, Gebel J, Engelhart S, Hartemann P (2005). Prevention and control of health care-associated waterborne infections in health care facilities. Am J Infect Control.

[8] Sehulster L, Chinn R, Arduino M, J C, Donlan R, Ashford D (2004). Guidelines for environmental infection control in health-care facilities. Recommendations from CDC and the Healthcare Infection Control Practices Advisory Committee (HICPAC).

[9] Ministère de la Santé et des Solidarités (2005). L’eau dans les établissements de santé - Guide technique.

[10] Batté M, Appenzeller B, Grandjean D, Fass S, Gauthier V, Jorand F (2003). Biofilms in drinking water distribution systems. Rev Environ Sci Bio.

[11] Société Française de Microbiologie (2013). Comité de l'antibiogramme de la société Française de microbiologie - Recommandations 2013.

[12] Masaoud E, Stryhn H (2010). A simulation study to assess statistical methods for binary repeated measures data. Prev Vet Med.

[13] Hu FB, Goldberg J, Hedeker D, Flay BR, Pentz MA (1998). Comparison of population-averaged and subject-specific approaches for analyzing repeated binary outcomes. Am J Epidemiol.

[14] Cui J (2008). QIC: Stata module to compute model selection criterion in GEE analyses.

[15] StataCorp LP (2007). Stata/SE 10.0.

[16] Battin TJ, Kaplan LA, Newbold JD, Cheng X, Hansen C (2003). Effects of current velocity on the nascent architecture of stream microbial biofilms. Appl Environ Microbiol.

